# Basic life support training for single rescuers efficiently augments their willingness to make early emergency calls with no available help: a cross-over questionnaire survey

**DOI:** 10.1186/2052-0492-2-28

**Published:** 2014-04-24

**Authors:** Keiko Hirose, Miki Enami, Hiroki Matsubara, Takahisa Kamikura, Yutaka Takei, Hideo Inaba

**Affiliations:** Department of Emergency Medical Science, Kanazawa University Graduate School of Medicine, 13-1 Takaramachi, Kanazawa, 920-8641 Japan; Department of Medical Science and Technology, Hiroshima International University, Hiroshima, Japan

**Keywords:** Emergency call, Basic life support, Willingness, Bystander, Training course

## Abstract

**Background:**

The aim of this study was to investigate effects of basic life support (BLS) training on willingness of single rescuers to make emergency calls during out-of-hospital cardiac arrests (OHCAs) with no available help from others.

**Methods:**

A cross-over questionnaire survey was conducted with two questionnaires. Questionnaires were administered before and after two BLS courses in fire departments. One questionnaire included two scenarios which simulate OHCAs occurring in situations where help from other rescuers is available (Scenario-M) and not available (Scenario-S). The conventional BLS course was designed for multiple rescuers (Course-M), and the other was designed for single rescuers (Course-S).

**Results:**

Of 2,312 respondents, 2,218 (95.9%) answered all questions and were included in the analysis. Although both Course-M and Course-S significantly augmented willingness to make early emergency calls not only in Scenario-M but also in Scenario-S, the willingness for Scenario-M after training course was significantly higher in respondents of Course-S than in those of Course-M (odds ratio 1.706, 95% confidential interval 1.301–2.237). Multiple logistic regression analysis for Scenario-M disclosed that post training (adjusted odds ratio 11.6, 95% confidence interval 7.84–18.0), age (0.99, 0.98–0.99), male gender (1.77, 1.39–2.24), prior BLS experience of at least three times (1.46, 1.25–2.59), and time passed since most recent training during 3 years or less (1.80, 1.25–2.59) were independently associated with willingness to make early emergency calls and that type of BLS course was not independently associated with willingness. Therefore, both Course-M and Course-S similarly augmented willingness in Scenario-M. However, in multiple logistic regression analyses for Scenario-S, Course-S was independently associated with willingness to make early emergency calls in Scenario-S (1.26, 1.00–1.57), indicating that Course-S more efficiently augmented willingness. Moreover, post training (2.30, 1.86–2.83) and male gender (1.26, 1.02–1.57) were other independent factors associated with willingness in Scenario-S.

**Conclusions:**

BLS courses designed for single rescuers with no help available from others are likely to augment willingness to make early emergency calls more efficiently than conventional BLS courses designed for multiple rescuers.

## Background

Survival after out-of-hospital cardiac arrests (OHCAs) depends on adherence to stages of the ‘chain of survival’. These include immediate recognition of cardiac arrest and activation of the emergency response system, early cardiopulmonary resuscitation (CPR), rapid defibrillation, effective advanced life support and integrated post-cardiac arrest care [1–3]. In particular, appropriate and prompt performance of the first three stages is essential for survival after OHCA [4–6]. In fact, accumulating evidence indicates that long delays in making emergency calls are associated with poorer OHCA outcomes [7–9]. These delays may reduce the beneficial effects of dispatcher-assisted CPR (DA-CPR) [10–13] because bystanders more frequently initiate CPR according to DA-CPR than on their own initiative [14].

A majority of OHCAs occur at home [15–17] and are relatively isolated from the emergency medical service (EMS) system. Therefore, multiple rescuers are rarely present [18] and the recognition of cardiac arrest, and the activation of EMS systems is frequently delayed [9, 19]. Public education on the importance of making early emergency calls may be effective in reducing this delay [20]. However, flowcharts in BLS guidelines and textbooks are basically designed for OHCAs witnessed by multiple rescuers [1–3, 21]. Therefore, the situations for which most BLS courses provide training are ideally those where help from others are easily available.

This study investigated the effects of BLS courses for single rescuers on their willingness to make emergency calls as lone bystanders. Because BLS courses for citizens are most frequently held in fire departments in Japan [22], we designed and conducted this study in co-operation with fire departments in our community.

## Methods

Data were collected in accordance with the national ethics guidelines for epidemiological surveys [23]. The study was approved by the review board of Kanazawa University Graduate School of Medicine (reference number: 924) (Table 1).

**Table 1:** **Study groups and design**

	Study groups			
	M-M/S	M-S/M	S-M/S	S-S/M
Type of instruction	For multiple rescuers		For single rescuer	
Type of scenarios in questionnaires				
Pre-training questionnaires	Scenario-M	Scenario-S	Scenario-M	Scenario-S
Post-training questionnaires	Scenario-S	Scenario-M	Scenario-S	Scenario-M
Study terms				
In central regions	2010.7.1–2010.9.30	2010.10.1–2010.12.31	2011.1.1–2011.3.31	2011.4.1–2011.6.30
In non-central regions	2011.1.1–2011.3.31	2011.4.1–2011.6.30	2010.7.1–2010.9.30	2010.10.1–2010.12.31

Scenario-M, in cases with multiple rescuers; Scenario-S, in cases with a single rescuer.

We conducted this investigation in co-operation with eight fire departments of the Ishikawa Prefecture. All eight fire departments were divided into two groups according to their location (three in central and four in non-central regions). The study period was from 1 July 2010 to 30 June 2011 and was divided into four terms. In each term, participants in BLS courses held by each fire department were assigned in a cross-over manner to one of four study groups which were classified on the basis of types of course design and questionnaire scenarios: M-M/S, M-S/M, S-M/S, and S-S/M (Table 2).

**Table 2:** **Questionnaire and choice**

Scenarios-M	Choices	Scenarios-S	Choices
Scenario-M1:	(a) Call 119 by yourself*	Scenario-S1:	(a) Call 119 with your phone*
In the afternoon on Sunday, you found an unknown woman collapsed in a station. She was unresponsive and was breathing abnormally. Passersby crowded around you and the woman. What do you do first?	(b) Ask one of the passersby to call 119 and start chest compressions*	A 65-year-old man collapsed in front of you on an array in your residential area. He is unresponsive. You are not able to judge whether he is breathing or not. Nobody is around you. You have a cellular phone. What do you do first?	(b) Call your reliable friend or relative
	(c) Inquire a reliable person what you should do or discuss with others regarding what to do		(c) Go back home and report the event to your family
	(d) Call 119 only when you are requested		(d) Go to the nearest residency and ask for help
	(e) Look for a station staff first		(e) Keep on checking if he is breathing
	(f) Keep on observing the woman		(f) Call police
	(g) Leave the scene		(g) Start chest compressions and wait for someone
	(h) Other		(h) Other
Scenario-M2:	(a) Call 119 by yourself*	Scenario-S2	(a) Call 119*
You and many relatives were at a relative's home for a Buddhist memorial event. One of your family members complained of sudden chest pain and collapsed. He or she became unresponsive. One of your relative reported that she or he is not breathing and appears to be in cardiac arrest. What do you do first?	(b) Ask one of your relatives to call 119 and start chest compressions*	When you are alone at home, your uncle visited you. When you talked with him, he complained of sudden chest pain and collapsed. He is unresponsive and breathing abnormally. What do you do?	(b) Call your reliable friend or relative
	(c) Discuss with others regarding what to do		(c) Call his family
	(d) Transport him or her to a medical office or hospital		(d) Call a medical office or hospital
	(e) Keep on observing him or her		(e) Transport him to a medical office or hospital
	(f) Other		(f) Keep on observing
			(g) Start chest compressions

*Desirable action(s).

One type of BLS course was conventional and was designed for multiple rescuers (Course-M), whereas the other was designed for single rescuers (Course-S). In course-M, participants were given a BLS instruction predominantly in a public location where they can send someone to place an emergency call and another to find and bring an AED. In course-S, participants were principally trained to act as single rescuers in a place where no help from others is initially available; they were instructed to place an emergency call on their own with a mobile or cordless phone and leave the victim only when there is no other option. In both course, all participants were similarly educated for CPR and AED use. All instructors involved in this study were fire department staff and were qualified to instruct on BLS. They were informed of the study design and were given a standard instruction manual. Two types of questionnaire were administered to participants in different orders before and after the BLS instruction course. One questionnaire included two scenarios (Scenario-M1 and Scenario-M2) which simulate OHCA cases wherein help from others is available. The other included scenarios (Scenario-S1 and Scenario-S2) wherein no help is available or the respondent is a lone rescuer. These two types of questionnaires were administered in different orders before and after the BLS instruction course. For respondent background, the questionnaire included age, gender, residential area, occupation, prior BLS training experience, and the time since the most recent course.

### Data analysis

Data analyses were performed using JMP ver. 7 for Windows (SAS Institute). The effects of the BLS course type on the willingness to make early emergency calls were analyzed using univariate analysis, and the chi-square test was applied with and without Pearson's correction. The Kruskal-Wallis test was used for non-parametric comparisons. We used multiple regression models to confirm the effects of the BLS course type and to elucidate the factors associated with willingness to make early emergency calls. In all analyses, *p* < 0.05 indicated statistical significance. If reported, unadjusted and adjusted odds ratios (ORs) have been predominantly presented in tables.

## Results

### Number of respondents

Of 2,312 respondents, 2,218 answered all questions and were included in analyses. No significant differences were observed in the ratio of respondents analyzed to those unanalyzed among the four study terms or between the two types of course or questionnaires (Table 3).

**Table 3:** **Comparisons of backgrounds among the four respondent groups**

		Group of respondents				***p*** value
		Common instructions for multiple rescuers ***N*** = 885		Specialized instruction for single rescuer ***N*** = 1,333		
		M-M/S	M-S/M	S-M/S	S-S/M	
		***N*** = 609	***N*** = 276	***N*** = 832	***N*** = 501	
Age, Median		41 (33–52)	45 (36–52)	42 (30–54)	40 (31–52)*	*p* = 0.007
Gender, % (*N*)	Male	34% (210)	66% (182)	52% (431)*	50% (249)*	*p* < 0.0001
	Female	66% (399)	34% (94)	48% (401)*	50% (252)*	
Residential area, % (*N*)	Central	85% (520)	37% (101)	20% (170)*	36% (179)	*p* < 0.0001
	Rural	14% (84)	61% (168)	78% (646)*	62% (311)	
	Other	1% (5)	3% (7)	2% (16)*	2% (11)	
Occupation, % (*N*)	Unemployed	37% (228)	37% (103)	24% (199)*	35% (175)	*p* < 0.0001
	Employed	63% (381)	63% (173)	76% (633)*	65% (326)	
Prior BLS training experience, % (*N*)	None	26% (159)	32% (87)	25% (210)	27% (137)	*p* = 0.659
	One time	34% (207)	30% (87)	32% (269)	32% (162)	
	Two times	20% (120)	18% (49)	22% (185)	22% (106)	
	Three times or more	20% (123)	21% (57)	20% (168)	19% (96)	
Time from most recent course	3 years or less	54% (244/450)	69% (131/190)	66% (413/626)*	60% (218/364)*	*p* = 0.0004
	More than 3 years	46% (206/450)	31% (59/190)	34% (213/626)*	40% (146/364)*	

*Significantly different from the corresponding group receiving common instructions for multiple rescuers (*p* < 0.05); Group S-M/S vs. Group M-M/S, Group S-S/M vs. Group M-S/M.

**Figure 1:**
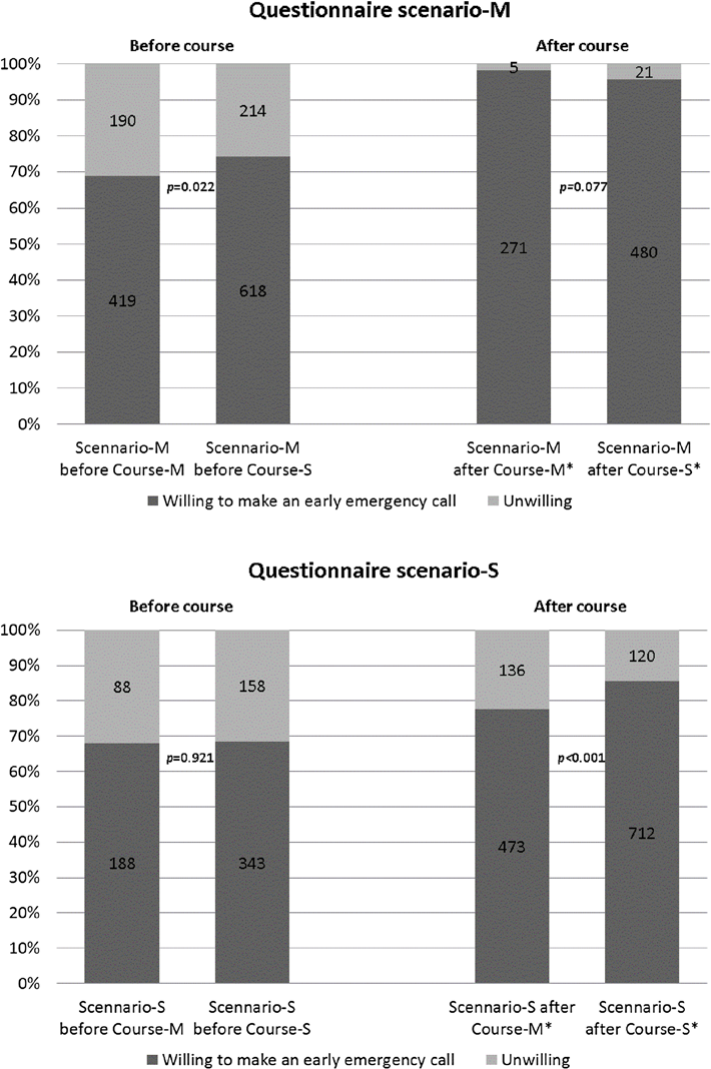
**Augmentation of willingness after the BLS training course.** Effects of Course-M and Course-S on willingness to make early emergency calls are individually presented in Scenario-M (*left panel*) and Scenario-S (*right panel*). * When analysis was made in each combination of BLS training course and scenario, both Course-M and Course-S significantly augmented willingness not only in Scenario-M but also in Scenario-S (*p* < 0.01).

No significant differences were observed with regard to prior BLS experience among the four groups. However, significant differences were observed with regard to age, gender, residential area, occupation and time passed since most recent course between the four respondent groups. Particularly, a large difference was observed with regard to residential area, with the majority of respondents from the central region in Group M-M/S and those from rural areas in the other three groups (Figure 1).

Effects of Course-M and Course-S on willingness to make early emergency calls are individually presented in Scenario-M (left panel) and Scenario-S (right panel). There was a small but significant differences in willingness between Course-M and Course-S in Questionnaire Scenario-M provided before the training courses (*p* = 0.022). However, there was a larger and significant difference in willingness in Scenario-S after the training courses (*p* < 0.001). The willingness to make early emergency calls significantly increased after both types of course; from 68.8% to 98.1% in Course M, from 74.3% to 95.8% (*p* < 0.01, shown by asterisks in Figure 1) (Table 4).

**Table 4:** **Univariate analysis followed by multiple logistic regression analysis**

Factors		Type of questionnaire scenarios					
		Scenario-M providing if-cases with multiple rescuers			Scenario-S providing if-cases with a single rescuer		
		Willing to make an early emergency call, % (***N***)	***p*** or odds ratio (95% CI) by univariate analysis	Adjusted odds ratio by multiple logistic regression analysis	Willing to make an early emergency call, % (***N***)	***p*** or odds ratio (95% CI) by univariate analysis	Adjusted odds ratio by multiple logistic regression analysis
Timing for questionnaire	Pre-training	72% (1,037/1,441)	Reference	Reference	68% (531/777)	Reference	Reference
	Post-training	97% (751/777)	*11.3 (7.49–16.9)*	*11.6 (7.84–18.0)*	82% (1,185/1,441)	*2.13 (1.75–2.63)*	*2.30 (1.86–2.83)*
Type of instruction course	Course-M (multiple rescuers)	78% (690/885)	Reference	Reference	75% (661/885)	Reference	Reference
	Course-S (single rescuer)	82% (1,098/1,333)	*1.32 (1.07–1.63)*	1.11 (0.83–1.49)	80% (1,055/1,333)	*1.29 (1.05–1.57)*	*1.26 (1.00–1.57)*
Age, years, median (25%–75%)	Unwilling	44 (35–54)	*p* = 0.005	*0.99 (0.98–0.99)*	44 (35–54)	*p* = 0.400	1.00 (0.99–1.00)
	Willing	41 (32–52)			41 (32–52)		
Gender	Female	76% (866/1,146)	Reference	Reference	76% (868–1,146)	Reference	Reference
	Male	86% (922/1,072)	*1.99 (1.60–2.48)*	*1.77 (1.39–2.24)*	79% (848–1,072)	1.21 (0.99–1.48)	*1.26 (1.02–1.57)*
Residential area, % (*N*)	Central	77% (970/750)	Reference	Reference	75% (730/970)	Reference	Reference
	Others	83% (1,038/1,248)	*1.45 (1.17–1.79)*	1.25 (0.93–1.67)	79% (986/1,248)	*1.24 (1.01–1.51)*	1.20 (0.95–1.50)
Occupation	Unemployed	78% (550/705)	Reference	Reference	75% (525/705)	Reference	Reference
	Employed	82% (1,238/1,513)	*1.27 (1.02–1.58)*	1.22 (0.95–1.57)	79% (1,191/1513)	*1.27 (1.03–1.56)*	1.09 (0.87–1.37)
Prior BLS training experience	None	76% (450/593)	*p* = 0.002	Reference	77% (454/593)	*p* = 0.461	Reference
	One time	80% (579/721)		1.17 (0.80–1.73)	77% (556/721)		1.02 (0.72–1.46)
	Two times	83% (382/460)		1.32 (0.89–1.96)	80% (368/460)		1.10 (0.77–1.58)
	Three times or more	85% (377/444)		*1.46 (1.25–2.59)*	76% (338/444)		0.65 (0.42–1.02)
Duration from most recent course	None	76% (450/593)	*p* = 0.001	Reference	77% (454/593)	*p* = 0.008	Reference
	3 years or less	85% (852/1,003)		*1.80 (1.25–2.59)*	80% (804/1,003)		1.30 (0.94–1.79)
	More than 3 years	78% (486/622)		1.04 (0.72–1.49)	74% (458/622)		0.86 (0.62–1.19)

For factors associated with willingness of respondents to make early emergency calls during Scenarios-S and -M; CI, confidence interval. Odds ratios in italics indicate significant differences.

We assessed respondent willingness to make early emergency calls as desirable choices in both questionnaire scenarios (Scenario-M and Scenario-S). We judged that respondents had willingness when they selected desirable action(s) in all of each two questionnaire scenarios. Univariate analyses revealed that willingness was significantly augmented by Course-S, which was specialized for single rescuers (unadjusted OR = 1.32), the post-training questionnaire (unadjusted OR = 11.3), younger age (*p* < 0.005), male gender (unadjusted OR = 1.99), employment status (unadjusted OR = 1.27), region other than central area (unadjusted OR = 1.45), prior BLS training experience (*p* = 0.002) and the time passed since the most recent training course (*p* = 0.001). However, multiple logistic regression analysis revealed that post-training (adjusted OR = 11.6), younger age (unit OR for age = 0.99), male gender (adjusted OR = 1.77), prior BLS training experience of three times or more (adjusted OR = 1.46, no experience as reference) and time from most recent training course being 3 years or less (adjusted OR = 1.80) were significantly associated with willingness of Scenario-M questionnaire respondents to make early emergency calls. On the other hand, the type of instruction was not an independent factor (Table 4).

Compared with the Scenario-M questionnaire, univariate analysis disclosed fewer factors related to willingness in the Scenario-S questionnaire. Willingness was significantly augmented by course-S, which was specialized for single rescuers (unadjusted OR = 1.29), the post-training questionnaire (unadjusted OR = 2.13), regions other than the central area (unadjusted OR = 1.24) and employment status (unadjusted OR = 1.27). However, prior BLS training experience did not significantly influence willingness. Multiple logistic regression analysis of responses to the Scenario-S questionnaire revealed that post-training (adjusted OR = 2.30), training Course-S (adjusted OR = 1.26) and male gender (adjusted OR = 1.26) were significantly associated with willingness to make early emergency calls.

## Discussion

Delay of emergency calls is a preventable human factor which is associated with poor OHCA outcomes [7, 8, 18]. A majority of previous reports regarding education and public awareness of BLS investigate the effects of BLS education on citizens' awareness and willingness to perform bystander CPR [24–26] and the quality of CPR [27–29]. Therefore, to the best of our knowledge, this cross-over questionnaire survey is the first to assess the effects of BLS training on attitudes toward making early emergency calls and to compare two types of BLS training course that are classified by the number of rescuers present.

We analyzed factors associated with willingness to make early emergency calls by comparing responses to scenarios wherein help from others is easily available or multiple rescuers are present (Scenario-M questionnaire) with scenarios wherein help is unavailable (Scenario-S questionnaire). In univariate analysis shown in Figure 1, the willingness for Scenario-S questionnaire after training course was significantly higher in participants of Course-S than in those of Course-M (odds ratio 1.706, 95% confidential interval 1.301–2.237), suggesting that Course-S more efficiently augmented willingness than Course-M. Multiple logistic regression analyses of Scenario-M questionnaire responses indicated that while post-course respondents were associated with willingness, BLS course types (Course-M or Course-S) were not, suggesting that both types of BLS training course potentially augment willingness to make emergency calls in situations where multiple rescuers were present. Moreover, prior BLS training course experience within 3 years was an independent factor. In contrast, multiple logistic regression analysis of responses to the Scenario-S questionnaire revealed that post-course respondents and BLS single rescuer Course-S were independent factors associated with willingness and that prior BLS training experience was not. These results indicate that the standard BLS training course for multiple rescuers does not augment willingness to make early emergency calls when the respondent is a single rescuer. Furthermore, other independent factors associated with willingness to make early emergency calls were younger age, male gender, and employment status.

Most OHCAs occur in homes [30, 31] at relatively isolated locations from the emergency medical service system, where emergency calls are frequently delayed [9, 19] and multiple rescuers are rarely present [18, 30]. Elderly family members have the highest probability of being a victim or a bystander of at-home OHCAs [9, 30], and their cardiac arrests are most frequently witnessed or recognized by spouses and daughters [32, 33]. The present study and our previous large questionnaire surveys [34, 35] have indicated that elderly and female citizens were more reluctant to place early emergency calls, presumably because of emotional stress [36, 37] and the large gender gap in Japan [38]. When these bystanders witness and recognize cardiac arrests as single bystanders or rescuers, high emotional stress leads to placement of the first call to reliable family members, relatives, friends or general practitioners [9, 34, 35]. Further, bystanders more frequently initiate CPR in compliance with DA-CPR than on their own initiative [10–13]. Therefore, during the training course-S for single rescuers, instructors emphasized the importance of making early emergency calls to get an advice from the dispatcher for resuscitation, although availability of DA-CPR was known to all participants in both training courses. It is likely that the BLS training course designed for single rescuers should be applied for elderly and female participants whose daily life is spent at home.

DA-CPR has been reported to increase the incidence of bystander CPR and is expected to improve the outcomes for individuals who experience OHCAs [14]. Strong recommendations for DA-CPR have been made by the International Liaison Committee on Resuscitation in their 2010 Consensus [39] as well as in a scientific statement from the American Heart Association [40]. However, the benefits of DA-CPR for OHCA outcomes are diminished by delay of emergency calls, which consequently delays bystander CPR. In addition to emphasis on early emergency calls in all BLS training courses, application of BLS training for single rescuers may diminish the delay in placing an emergency call.

This study had several limitations. Two BLS courses (Course-M and Course-S) were performed in a cross-over manner in eight fire departments of the Ishikawa Prefecture. Multiple logistic regression analyses revealed that residential areas or locations of fire departments were not independent factors associated with willingness to make early emergency calls (Table 4). However, we did not evaluate the quality of BLS instructions. Moreover, although all instructors involved in this study were qualified staff adhering to standard instruction manuals, the quality of instruction may have affected the results of this study. Approximately half of the respondents were female. A majority of respondents were middle aged, were employed and had previous BLS training experience. Therefore, the results of this study may not reflect the willingness of elderly females, who are the most likely witnesses of cardiac arrests. Because of limitations of time, the questionnaire in this study was only designed to assess willingness to make early emergency calls. The willingness to perform other BLS actions, including CPR and use of automated external defibrillators, were not evaluated. The effects of two types of BLS training were evaluated by comparing answers to questionnaires administered before the BLS course with those administered immediately after the BLS course. Therefore, the duration of willingness to make early emergency calls remains unknown.

## Conclusions

In contrast with the conventional BLS course for multiple rescuers, the BLS course for single rescuers is likely to efficiently augment willingness to make early emergency calls when participants are single rescuers of OHCA patients. In addition to emphasis on early emergency calls in all BLS training courses, application of BLS training for single rescuers may diminish the delay in placing an emergency call.
